# Compulsive Buying Behavior: Clinical Comparison with Other Behavioral Addictions

**DOI:** 10.3389/fpsyg.2016.00914

**Published:** 2016-06-15

**Authors:** Roser Granero, Fernando Fernández-Aranda, Gemma Mestre-Bach, Trevor Steward, Marta Baño, Amparo del Pino-Gutiérrez, Laura Moragas, Núria Mallorquí-Bagué, Neus Aymamí, Mónica Gómez-Peña, Salomé Tárrega, José M. Menchón, Susana Jiménez-Murcia

**Affiliations:** ^1^Ciber Fisiopatología Obesidad y Nutrición (CIBERObn), Instituto de Salud Carlos IIIBarcelona, Spain; ^2^Departament de Psicobiologia i Metodologia de les Ciències de la Salut, Universitat Autònoma de BarcelonaBarcelona, Spain; ^3^Pathological Gambling Unit, Department of Psychiatry, Bellvitge University Hospital-IDIBELLBarcelona, Spain; ^4^Department of Clinical Sciences, Faculty of Medicine, University of BarcelonaBarcelona, Spain; ^5^Nursing Department of Mental Health, Public Health, Maternal and Child Health, Nursing School, University of BarcelonaBarcelona, Spain; ^6^Ciber de Salud Mental (CIBERSAM), Instituto de Salud Carlos IIIBarcelona, Spain

**Keywords:** behavioral addictions, compulsive buying behavior, gambling disorder, internet gaming disorder, internet addiction, sex addiction

## Abstract

Compulsive buying behavior (CBB) has been recognized as a prevalent mental health disorder, yet its categorization into classification systems remains unsettled. The objective of this study was to assess the sociodemographic and clinic variables related to the CBB phenotype compared to other behavioral addictions. Three thousand three hundred and twenty four treatment-seeking patients were classified in five groups: CBB, sexual addiction, Internet gaming disorder, Internet addiction, and gambling disorder. CBB was characterized by a higher proportion of women, higher levels of psychopathology, and higher levels in the personality traits of novelty seeking, harm avoidance, reward dependence, persistence, and cooperativeness compared to other behavioral addictions. Results outline the heterogeneity in the clinical profiles of patients diagnosed with different behavioral addiction subtypes and shed new light on the primary mechanisms of CBB.

## Introduction

Compulsive buying behavior (CBB), otherwise known as shopping addiction, pathological buying or compulsive buying disorder, is a mental health condition characterized by the persistent, excessive, impulsive, and uncontrollable purchase of products in spite of severe psychological, social, occupational, financial consequences (Müller et al., 2015b). Whereas, ordinary non-addicted consumers state value and usefulness as their primary motives for shopping, compulsive buyers make purchases in order to improve their mood, cope with stress, gain social approval/recognition, and improve their self-image (Lejoyeux and Weinstein, 2010; Karim and Chaudhri, 2012; McQueen et al., 2014; Roberts et al., 2014). Although the aftermath of protracted CBB includes feelings of regret/remorse over purchases, shame, guilt, legal and financial problems, and interpersonal difficulties, people with CBB fail in their attempts to stop compulsive buying (Konkolý Thege et al., 2015).

The frequency of CBB has increased worldwide during the two last decades. A recent meta-analysis estimated a pooled prevalence of 4.9% for CBB in adult representative samples, with higher ratios for university students, those of non-community origin and shopping-specific participants (Maraz et al., 2015). However, prevalence estimations in epidemiological research vary and can range from 1 to 30% depending on the type of sample studied (Basu et al., 2011).

One major difficulty in estimating CBB prevalence is that the categorization of this psychopathological condition in international classification systems continues to be debated and consensus on diagnosis criteria has yet to be reached. As a matter of fact, the concept of “addiction” itself was a contentious subject matter in the preparation of the Diagnostic and Statistical Manual of Mental Disorders fifth edition (DSM-5; American Psychiatric Association, 2013; Piquet-Pessôa et al., 2014). Currently the available operational definitions for CBB have relied on similarities with disorders in the impulsive control spectrum (Potenza, 2014; Robbins and Clark, 2015), mainly linked to substance use disorders (Grant et al., 2013), obsessive-compulsive disorder (Weinstein et al., 2015), eating disorders (Fernández-Aranda et al., 2006, 2008; Jiménez-Murcia et al., 2015) and other behavioral addictions such as gambling disorder (Black et al., 2010), Internet gaming disorder (IGD) and Internet addiction (Suissa, 2015; Trotzke et al., 2015), and sexual addiction (Derbyshire and Grant, 2015; Farré et al., 2015).

The specific etiology of CBB is still unknown. Diverse factors have been proposed as likely contributors and the few CBB studies conducted to date have largely been centered on neurobiological factors, with research on genetic factors and CBB being nonexistent. As in substance use disorders, brain imaging studies in people with CBB and other behavioral addictions have consistently found abnormalities in frontoparietal regions, reward processing, and limbic systems (Raab et al., 2011; Baik, 2013; Leeman and Potenza, 2013; Probst and van Eimeren, 2013; Vanderah and Sandweiss, 2015). However, the presently available neurological evidence does not fully explain how concrete neural mechanisms and cognitive processes can cause normal-shopping behavior to become addictive in the absence of exogenous drug stimulation (Clark, 2014; Engel and Caceda, 2015). Unlike in other addictive conditions, it has been stated that the development of CBB depends on the presence of particular cultural mechanisms, such as a market-based economy, a wide variety of available goods, disposable income, and materialistic values (Unger et al., 2014).

Regarding the CBB phenotype, research studies highlight shared common features with other behavioral addictions (El-Guebaly et al., 2012; Choi et al., 2014; Grant and Chamberlain, 2014; Di Nicola et al., 2015). Gray's Reinforcement Sensitivity Theory, which has been applied to other behavioral addictive disorders, argues that high levels of behavioral approach system (BAS) predispose individuals to engage in impulsive behaviors (Franken et al., 2006). It has also been used to explain the addictive processes underlying CBB: both reinforcement-punishment systems seem to participate in the onset and development of this disorder (Davenport et al., 2012). Although in clinical samples, a greater association has been found between this disorder and higher levels of behavioral activation (Claes et al., 2010; Müller et al., 2014). Furthermore, dysfunctional emotion regulation also seems to be implied in the phenotype of behavioral addictions, particularly in aspects such as managing cravings and withdrawal symptoms(Kellett et al., 2009; Williams and Grisham, 2012).

The early onset of problematic behavior is also considered a common feature of these addictive activities, and epidemiological research has found that addictive behaviors tend to become problematic in late adolescence (Balogh et al., 2013; Maraz et al., 2015). It is during this stage of development when impulsivity and risky behaviors may be most socially tolerated or even promoted by peers, which could constitute a potential risk factor for developing an addiction (Dayan et al., 2010; Hartston, 2012). It must be highlighted however that some representative surveys in Europe in the recent years have demonstrated increases in the estimated prevalence of behavioral addictions in older adult populations (Mueller et al., 2010).

The study of the CBB phenotype and related personality traits has also generated consistent results with other behavioral addictions. Research has shown that compulsive buying is characterized by high impulsivity scores, novelty seeking and compulsivity (Black et al., 2012; Di Nicola et al., 2015; Munno et al., 2015), along with high levels in both positive and negative urgency traits (Rose and Segrist, 2014), coinciding with the findings obtained in gambling disorder (Janiri et al., 2007; Tárrega et al., 2015), IGD or in sexual addictions (Jiménez-Murcia et al., 2014b; Farré et al., 2015).

Finally, CBB is associated with significant comorbidity, particularly with psychiatric conditions that are also highly prevalent in other behavioral addictions (Mueller et al., 2010; Aboujaoude, 2014), such as mood disorders, anxiety disorders, substance use, other impulse control disorders, and eating disorders (Fernández-Aranda et al., 2006, 2008).

Heterogeneous features in both clinical and personality aspects have also been reported when comparing CBB with other behavioral addictions. Firstly, epidemiological studies point to strong sex differences (Fattore et al., 2014): whereas CBB is more prevalent in women (Otero-López and Villardefrancos, 2014), gambling disorder (Ashley and Boehlke, 2012), and sexual addiction (Farré et al., 2015) are more prevalent in men.

Regarding CBB patients' psychopathological state, to our knowledge few studies with clinical samples have assessed the specific differences between CBB and other behavioral additions. As such, the objectives of this study are: (a) to ascertain the most relevant socio-demographic and clinical characteristics associated to CBB in a large clinical sample of patients with behavioral addictions; and (b) to compare the CBB profile with other behavioral addictions (sexual addiction, IGD, Internet addiction, and gambling disorder).

## Materials and methods

### Sample

All the patients who arrived at the Pathological Gambling Unit in the Psychiatry Department at Bellvitge University Hospital in Barcelona (Spain), from January 2005 to August 2015, were potential participants in this study. Exclusion criteria for the study were the presence of an organic mental disorder, intellectual disability, or active psychotic disorder. Bellvitge University Hospital is a public hospital certified as a tertiary care center for the treatment of behavioral addictions and oversees the treatment of highly complex cases. The catchment area of the hospital includes over two million people in the Barcelona metropolitan area.

All participants were diagnosed according to DSM-IV criteria (SCID-I; First et al., 1996) and using specific questionnaires for each disorder. Interviews were conducted by psychologists and psychiatrists with more than 15 years of experience in the field.

The study sample included *n* = 3324 patients, who were classified into five groups according to their diagnostic subtype: CBB (*n* = 110), sexual addiction (*n* = 28), IGD (*n* = 51), Internet addiction (*n* = 41), and gambling disorder (*n* = 3094). Mutual exclusivity criterion was required to include the patients in the groups, that is, the addictions considered in this study did not occur at the same time to allow for the estimation and comparison of the specific clinical state of each behavioral addiction type (39 patients were excluded from our analyses for meeting the criteria of having more than one behavioral addiction).

### Measures

#### Evaluation of current and lifetime substance use disorders and impulsive related behaviors

Patients were assessed using a structured clinical face-to-face interview modeled after the Structured Clinical Interview for DSM-IV (SCID-I; First et al., 1996), covering the lifetime presence of impulsive behaviors, namely alcohol and drug abuse, comorbid impulse control disorders (such as CBB, sexual addiction, and IGD and Internet addiction).

#### Diagnostic questionnaire for pathological gambling according to DSM criteria (Stinchfield, 2003)

This 19-item questionnaire allows for the assessment of DSM-IV (American Psychiatric Association, 1994) diagnostic criteria for pathological gambling (in the present study called GD). Convergent validity with the SOGS scores in the original version was very good [*r* = 0.77 for representative samples and *r* = 0.75 for gambling treatment groups (Stinchfield, 2003)]. Internal consistency in the Spanish adaptation used in this study was α = 0.81 for the general population and α = 0.77 for gambling treatment samples (Jiménez-Murcia et al., 2009). In this study, the total number of DSM-5 criteria for GD was analyzed. Cronbach's alpha in the sample was very good (α = 0.81).

#### South oaks gambling screen (SOGS) (Lesieur and Blume, 1987)

This self-report, 20-item, screening questionnaire discriminates between probable pathological, problem, and non-problem gamblers. The Spanish validated version used in this study has shown excellent internal consistency (α = 0.94) and test-retest reliability (*r* = 0.98; Echeburúa et al., 1994). Consistency in the sample of this work was adequate (α = 0.76).

#### Diagnostic criteria for compulsive buying according to Mcelroy et al. (1994)

These criteria have received wide acceptance in the research community, although their reliability and validity have not yet been determined (Tavares et al., 2008). It's worth noting that no formal diagnostic criteria for CBB have been accepted for the DSM or the ICD−10. At present, it is recommended that CBB diagnosis be determined via detailed face−to−face interviews which explore “buying attitudes, associated feelings, underlying thoughts, and the extent of preoccupation with buying and shopping” (Müller et al., 2015b).

#### Diagnostic criteria for IGD according to Griffiths and Hunt (1995, 1998)

To assess IGD diagnosis and to establish the level of dependence on video games, clinical experts conducted a clinical face-to-face interview considering the scale designed by Griffiths and Hunt (1995, 1998). This interview evaluated aspects such as the frequency of the problematic behavior, the interference generated in daily functioning because of maladaptive use of video games or the presence of tolerance and difficulties in abstinence management.

#### Diagnostic criteria for sexual addiction according to DSM-IV-TR (American Psychiatric Association, 2000)

To assess sexual addiction, a battery of items was administered, which were based on the proposed definition in the DSM-IV-TR (American Psychiatric Association, 2000) in the Sexual Disorders Not Otherwise Specified section (302.9). In making our assessment, the following clinical description was given special weight: “distress about a pattern of repeated sexual relationship involving a succession of lovers who are experienced by the individual only as things to be used.”

#### Diagnostic criteria for internet addiction according to Echeburúa (1999)

To assess Internet addiction, a clinical interview that adapts the nine criteria from Echeburúa (1999) in yes/no responses was used. Four to six scores indicate a risk of dependency and 7–9 an already established problem. Internet addiction categorization is focused on excessive and continuous use of the Internet (social networking, watching videos, television series, and movies online, etc.). These items also explore the urge to carry out this behavior or the failed attempts to reduce its frequency.

#### Temperament and character inventory-revised (TCI-R) (Cloninger, 1999)

The TCI-R is a reliable and valid 240-item questionnaire which measures seven personality dimensions: four temperament (novelty seeking, harm avoidance, reward dependence, and persistence) and three character dimensions (self-directedness, cooperativeness, and self-transcendence). All items are measured on a 5-point Likert-type scale. The scales in the Spanish revised version showed adequate internal consistency (Cronbach's alpha α mean value of 0.87; Gutiérrez-Zotes et al., 2004). Cronbach's alpha (α) in the sample used in this study is in the good to excellent range (index for each scale is included in **Table 2**).

#### Symptom checklist-revised (SCL-90-R) (Derogatis, 1990)

The SCL-90-R evaluates a broad range of psychological problems and psychopathological symptoms. This questionnaire contains 90 items and measures nine primary symptom dimensions: somatization, obsession-compulsion, interpersonal sensitivity, depression, anxiety, hostility, phobic anxiety, paranoid ideation, and psychoticism. It also includes three global indices: (1) a global severity index (GSI), designed to measure overall psychological distress; (2) a positive symptom distress index (PSDI), to measure symptom intensity; and (3) a positive symptom total (PST), which reflects self-reported symptoms. The Spanish validation scale obtained good psychometrical indexes, with a mean internal consistency of 0.75 (Cronbach's alpha; Martínez-Azumendi et al., 2001). Cronbach's alpha (α) in the sample of this study is in the good to excellent range (indexes for each scale are included in **Table 2**).

#### Alcohol use disorders identification test (AUDIT) (Saunders et al., 1993)

This test was developed as a simple screening method for excessive alcohol consumption. AUDIT consists of 10 questions examining alcohol consumption levels, symptoms of alcohol dependence and alcohol-related consequences. Internal consistency has been found to be high, and rest-retest data have suggested high reliability (0.86) and sensitivity around 0.90; specificity in different settings and for different criteria averages 0.80 or more. Three categories were considered for this study, based on the ranges defined by Reinert and Allen (2002): null-low (raw scores under 6 for women and under 8 for men), abuse (raw scores between 6 and 20 for women and between 8 and 20 for men) and risk of dependence (raw scores above 20).

#### Additional data

Demographic, clinical, and social/family variables related to gambling were measured using a semi-structured, face-to-face clinical interview described elsewhere (Jiménez-Murcia et al., 2006). Some of the CBB behavior variables covered were the age of CBB onset, the mean and maximum monetary investment in a single shopping episode, and the total amount of accumulated debts.

### Procedure

The present study was carried out in accordance with the latest version of the Declaration of Helsinki. The University Hospital of Bellvitge Ethics Committee of Clinical Research approved the study, and signed consent was obtained from all participants. Experienced psychologists and psychiatrists conducted the two face-to-face clinical interviews.

### Statistical analysis

Statistical analysis was carried out with Stata13.1 for Windows. First, the comparison of the sociodemographical, clinical and personality measures between the derived empirical clusters was based on chi-square tests (χ^2^) for categorical variables and analysis of variance (ANOVA) for quantitative measures. Cohen's-*d* measured the effect size of pairwise comparisons (|*d*|> 0.50 was considered moderate effect size and |*d*|> 0.80 high effect size). Bonferroni-Finner's correction controlled for Type-I error due to multiple statistical comparisons for variables measuring clinical state.

Second, a multinomial model valued the capacity of the participants' sex, age, age of onset, education level, civil status, and personality traits levels to discriminate the presence of CBB compared to the other behavioral addictions (gambling, Internet, IGD, and sexual addiction). This model constitutes a generalization of the logistic regression to multiclass-nominal-criteria (dependent variables with more than two categorical levels). Its parameters are estimated to predict the probability of the different categories compared to a reference category-level. In this study, with the aim of obtaining a discriminative model for the presence of CBB, this diagnostic subtype was defined as the reference level. In addition, the set of independent variables was simultaneously included into the model to determine the specific contribution of each variable in identifying CBB. The global predictive capacity of the model was assessed using the McFadden pseudo-R^2^ coefficient.

Third, multiple regressions models valued the predictive capacity of the participants' sex, age, age of onset, and personality traits on the psychopathology symptom levels registered on the SCL-90-R depression, anxiety and GSI scales. The ENTER procedure was used to simultaneously include the set of predictors to obtain the specific contribution of each factor to symptom levels.

## Results

### Evolution of the prevalence of consultations for behavioral addictions

**Figure 1:**
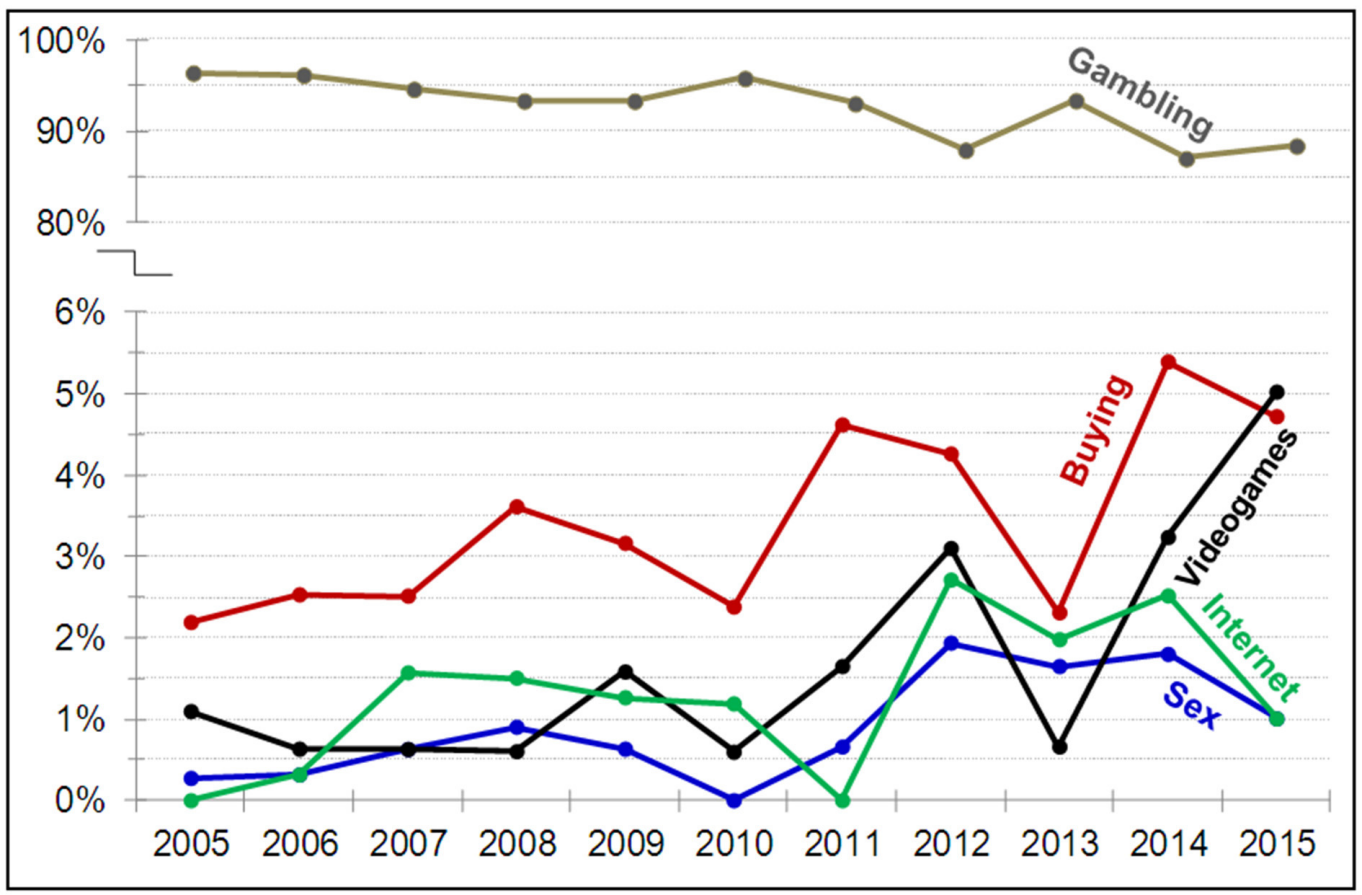
**Evolution of the prevalence of consultations due to different behavioral addictions**.

Figure 1 shows the prevalence of patients attending the specialized unit for treatment because of CBB in comparison to other behavioral addictions (gambling disorder, sexual addiction, IGD, or Internet addiction). The prevalence of consultations due to CBB increased from 2.48% in 2005 to 5.53% in 2015, obtaining a significant linear trend (χ^2^= 17.3, *df* = 1, *p* = 0.006) and no statistically significant deviation from linearity (χ^2^= 7.27, *df* = 9, *p* = 0.609). Our results demonstrate that the prevalence of gambling disorder was significantly higher compared to the other behavioral additions. As a whole, the prevalence of consultations was higher for CBB compared to IGD, Internet, and sexual addiction (except for IGD in 2015), but these differences were low.

### Comparison between CBB and the other behavioral additions

Table 1 contains the difference between diagnostic subtypes and the patients' sociodemographical variables, as well as data on substance abuse. The frequency of women in the CBB group (71.8%) was clearly higher when compared to the other diagnostic conditions (between 3.6% for sex addiction to 26.8% to Internet addiction). Considering other variables, CBB was characterized by: (a) a higher level of education compared to IGD and gambling addiction; (b) higher prevalence of being married or living with a partner compared to the IGD and Internet addiction groups; (c) higher levels of employment compared to IGD; and (d) compared to gambling disorder, lower prevalence of smoking, and alcohol abuse and other drug use/abuse.

**Table 1:** **Comparison between diagnostic subtypes for categorical variables: chi-square test and contrasts of buying subtype vs. the other diagnostic subtype**.

	**Proportions (%)**					**Group**			**Contrasts: buying vs. other addictions**							
	**Buying**	**Sex**	**Internet/gaming**	**Internet**	**Gambling**	**Chi-square tests**			**Sex**		**Internet/gaming**		**Internet**		**Gambling**	
	***n = 110***	***n = 28***	***n = 51***	***n = 41***	***n = 3.094***	**χ^2^**	***df***	***p***	***p***	***|d|***	***P***	***|d|***	***p***	***|d|***	***p***	***|d|***
**SEX**																
Female	71.8	3.6	5.9	26.8	10.1	387.15	4	<0.001^*^	0.001^*^	**1.98**^**†**^	0.001^*^	**1.84**^**†**^	0.001^*^	**1.01**^**†**^	0.001^*^	**1.61**^**†**^
Male	28.2	96.4	94.1	73.2	89.9											
**ORIGIN**																
Immigrant	1.8	0	3.9	2.4	6.5	7.41	4	0.131	0.472	0.19	0.425	0.13	0.808	0.04	0.100	0.24
Spanish	98.2	100	96.1	97.6	93.5											
**EDUCATION**																
Primary	33.7	26.9	40.0	32.5	57.8	88.61	8	<0.001^*^	0.778	0.15	0.022^*^	0.13	0.291	0.02	0.001^*^	**0.50**^**†**^
Secondary	43.3	50.0	55.6	55.0	36.3					0.14		0.25		**0.24**		0.14
University	23.1	23.1	4.4	12.5	5.9					0.00		**0.56**^**†**^		0.28		**0.50**^**†**^
**CIVIL STATUS**																
Single	35.5	22.2	91.8	65.0	35.4	84.98	8	<0.001^*^	0.260	0.30	0.001^*^	**1.44**^**†**^	0.005^*^	**0.62**^**†**^	**0.962**	0.00
Married-couple	49.5	51.9	6.1	30.0	50.5					0.05		**1.11**^**†**^		0.41		0.02
Divorced	15.0	25.9	2.0	5.0	14.1					0.27		0.48		0.34		0.03
**EMPLOYED**																
No	50.0	35.7	79.6	56.1	43.5	30.00	4	<0.001^*^	0.177	0.29	0.001^*^	**0.65**^**†**^	0.506	0.12	0.183	0.13
Yes	50.0	64.3	20.4	43.9	56.5											
**SMOKE USE**																
No	62.7	67.9	76.5	75.6	38.7	83.36	4	<0.001^*^	0.614	0.11	0.084	0.30	0.137	0.28	0.001^*^	0.49
Yes	37.3	32.1	23.5	24.4	61.3											
**AUDIT**																
Low	95.4	85.7	98.0	95.1	85.0	19.19	8	0.018^*^	0.065	0.34	0.415	0.15	0.940	0.01	0.010^*^	0.36
Abuse	4.6	14.3	2.0	4.9	14.3					0.34		0.15		0.01		0.34
Risk dependence	0	0	0	0	0.7					0.00		0.00		0.00		0.12
**OTHER DRUGS**																
No	97.2	85.7	92.2	95.0	90.9	6.97	4	0.138	0.014^*^	0.42	0.146	0.23	0.506	0.12	0.024^*^	0.27
Yes	2.8	14.3	7.8	5.0	9.1											

* *Bold, significant comparison (0.05 level)*.

† *Bold: effect size in the moderate (|d|> 0.50) to high (|d|> 0.80) range. p-values include Bonferroni-Finner correction*.

Table 2 includes mean comparisons between CBB and other diagnostic subtypes for the variables measuring clinical state: patients' age, age of onset, and duration of the problematic behaviors, psychopathological symptoms (SCL-90-R scales) and personality traits (TCI-R scales). No statistical differences emerged comparing CBB with the sexual addiction group. Compared to IGD, Internet addiction and gambling disorder, the CBB clinical profile was characterized by: (a) higher mean age and age of onset compared to IGD and Internet addiction; (b) as a whole, higher psychopathological symptoms (many SCL-90-R scales obtained higher mean scores); and (c) higher mean scores in the personality traits novelty seeking, harm avoidance (in comparison with gambling disorder), reward dependence (in comparison with IGD and gambling disorder), persistence (in comparison with IGD and Internet addiction), and cooperativeness (in comparison with IGD and gambling disorder).

**Table 2:** **Comparison of clinical profiles between diagnostic subtypes at baseline: ANOVA and effect size for pairwise comparisons**.

		**Means Buying**	**Sex**	**Internet/gaming**	**Internet**	**Gambling**	**ANOVA**		**Contrasts: buying vs. other addictions**							
									**Sex**		**Internet/gaming**		**Internet**		**Gambling**	
		***n = 110***	***n = 28***	***n = 51***	***n = 41***	***n = 3.094***	***F_4;3319_***	***p***	***p***	***|d|***	***p***	***|d|***	***p***	***|d|***	***p***	***|d|***
Age (years)		43.3	41.3	22.0	31.7	42.9	38.03	<0.001^*^	0.909	0.17	0.001^*^	**2.15**^**†**^	0.001^*^	**0.96**^**†**^	0.997	0.03
Onset (years)		38.9	37.5	19.9	29.8	38.3	26.25	<0.001^*^	0.973	0.11	0.001^*^	**1.81**^**†**^	0.001^*^	**0.72**^**†**^	0.973	0.05
Duration (years)		4.4	4.3	2.5	2.4	4.9	3.82	0.013^*^	0.999	0.01	0.233	0.42	0.253	0.45	0.776	0.09
SCL-90-R: Somatization	*α = 0.89*	1.4	1.1	0.5	0.9	0.9	11.96	<0.001^*^	0.151	0.37	0.001^*^	**1.03**^**†**^	0.001^*^	**0.62**^**†**^	0.001^*^	**0.52**^**†**^
SCL-90-R: Obs./comp.	*α = 0.86*	1.8	1.5	1.1	1.5	1.1	16.99	<0.001^*^	0.406	0.25	0.001^*^	**0.79**^**†**^	0.193	0.31	0.001^*^	**0.68**^**†**^
SCL-90-R: Int. sensitivity	*α = 0.85*	1.4	1.3	1.1	1.1	1.0	6.63	<0.001^*^	0.880	0.14	0.135	0.30	0.138	0.35	0.001^*^	0.44
SCL-90-R: Depressive	*α = 0.90*	2.0	1.8	1.0	1.5	1.5	11.98	<0.001^*^	0.454	0.25	0.001^*^	**0.99**^**†**^	0.004^*^	**0.56**^**†**^	0.001^*^	**0.53**^**†**^
SCL-90-R: Anxiety	*α = 0.87*	1.5	1.3	0.8	1.0	1.0	9.81	<0.001^*^	0.776	0.16	0.001^*^	**0.77**^**†**^	0.006^*^	**0.53**^**†**^	0.001^*^	0.48
SCL-90-R: Hostility	*α = 0.82*	1.2	1.2	1.1	1.0	0.9	5.15	<0.001^*^	0.999	0.03	0.509	0.20	0.268	0.31	0.001^*^	0.37
SCL-90-R: Phobic	*α = 0.80*	0.8	0.6	0.3	0.5	0.5	6.93	<0.001^*^	0.168	0.36	0.001^*^	**0.61**^**†**^	0.018^*^	0.44	0.001^*^	0.42
SCL-90-R: Paranoid	*α = 0.77*	1.3	1.1	1.1	1.0	0.9	6.43	<0.001^*^	0.850	0.15	0.617	0.17	0.108	0.38	0.001^*^	0.43
SCL-90-R: Psychotic	*α = 0.83*	1.1	1.3	0.6	1.0	0.9	4.65	0.001^*^	0.512	0.23	0.004^*^	**0.56**^**†**^	0.855	0.14	0.065	0.22
SCL-90-R: GSI	*α = 0.98*	1.5	1.3	0.9	1.1	1.0	10.41	<0.001^*^	0.645	0.20	0.001^*^	**0.78**^**†**^	0.017^*^	0.49	0.001^*^	**0.53**^**†**^
SCL-90-R: PST	*α = 0.98*	54.0	50.7	37.2	48.0	46.3	5.53	<0.001^*^	0.895	0.14	0.001^*^	**0.79**^**†**^	0.416	0.28	0.002^*^	0.35
SCL-90-R: PSDI	*α = 0.98*	2.3	2.1	1.8	1.9	1.9	11.07	<0.001^*^	0.740	0.21	0.001^*^	**0.63**^**†**^	0.006^*^	**0.59**^**†**^	0.001^*^	**0.60**^**†**^
TCI-R: Novelty seeking	*α = 0.80*	114.4	108.2	103.0	101.5	108.8	8.13	<0.001^*^	0.154	0.42	0.001^*^	**0.85**^**†**^	0.001^*^	**0.91**^**†**^	0.001^*^	0.39
TCI-R: Harm avoidance	*α = 0.82*	109.7	103.7	102.8	105.8	101.3	6.05	<0.001^*^	0.341	0.32	0.089	0.34	0.617	0.20	0.001^*^	0.44
TCI-R: Reward depend.	*α = 0.77*	104.8	102.5	95.3	98.1	99.7	3.93	0.006^*^	0.902	0.14	0.002^*^	**0.55**^**†**^	0.073	0.39	0.005^*^	0.33
TCI-R: Persistence	*α = 0.87*	108.0	104.0	94.8	95.5	109.4	9.83	<0.001^*^	0.821	0.19	0.002^*^	**0.65**^**†**^	0.008^*^	**0.68**^**†**^	0.924	0.07
TCI-R: Self-directed.	*α = 0.85*	125.0	118.8	125.2	123.0	128.1	2.27	0.069	0.505	0.27	1.000	0.01	0.971	0.09	0.494	0.13
TCI-R: Cooperativen.	*α = 0.81*	137.1	128.3	128.6	132.4	132.0	2.76	0.037^*^	0.074	**0.52**^**†**^	0.025^*^	0.45	0.448	0.27	0.019^*^	0.30
TCI-R: Self-Trans.	*α = 0.83*	66.0	63.6	59.3	64.0	64.4	1.57	0.178	0.888	0.15	0.050	0.41	0.903	0.13	0.706	0.10

* *Bold, significant comparison (0.05 level)*.

† *Bold: effect size in the moderate (|d|> 0.50) to high (|d|> 0.80) range. α: Cronbach's-alpha for the scale in the sample. p-values include Bonferroni-Finner correction*.

**Figure 2:**
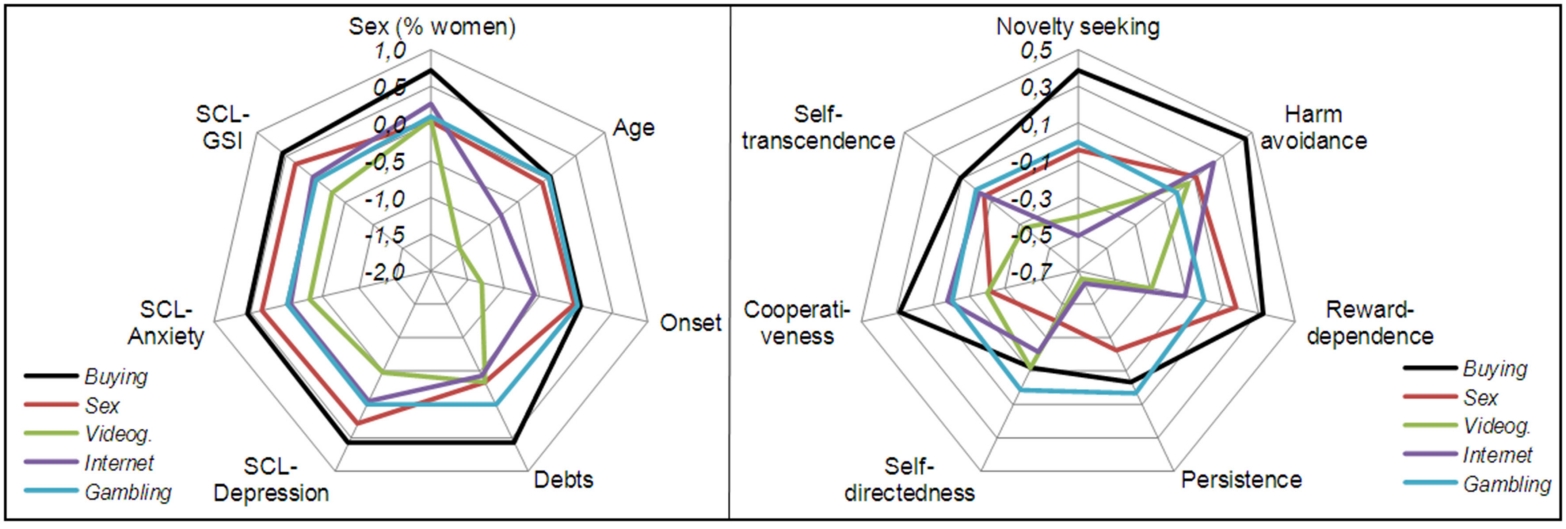
**Radiar-charts for the main clinical variables in the study and personality traits**.

Figure 2 includes two radar-charts to graphically summarize the clinical and personality profiles for the different diagnostic subtypes in the most relevant variables of the study. The percentage of women was plotted for gender distribution and the z-standardized scores in the own sample for the quantitative clinical measures (standardization was made due to the different ranges –minimum to maximum values– of these variables).

### Discriminative model for the presence of CBB compared to other behavioral addictions

Table 3 contains the results of the multinomial model measuring the discriminative capacity of patients' sex, age, age of onset, education level, marital status, and personality profile. Compared to all the other diagnostic subtypes, the probability of CBB is clearly higher in women and individuals with higher scores in the personality traits novelty seeking, harm avoidance and self-directedness. However, it should be noted that scores on self-directedness were in the clinically low range for all groups when considering general population normative scores. The opposite pattern emerges in the case of harm avoidance, in that all diagnostic groups were in the clinically high range, with those with CBB scoring the highest. In addition, older age is predictive of CBB compared to Internet and IGD, higher education levels increased the probability of CBB compared to gambling disorder, and moderate levels of persistence (rather than low) are more likely in CBB compared to Internet and IGD.

**Table 3:** **Discriminative capacity of age, age of onset, studies level, civil status, and personality profile in the presence of a diagnostic subtype (n = 3.324)**.

	**Likelihood ratio**		**Parameter estimates**															
	**tests**		**Gambling vs. buying**				**Internet vs. buying**				**Internet/gaming vs. buying**				**Sex vs. buying**			
	**χ^2^**	***p***	***p***	***OR***	**95%CI**		***p***	***OR***	**95%CI**		***p***	***OR***	**95%CI**		***p***	***OR***	**95%CI**	
Sex (male)	152.7	**<0.001**	**<0.001**	19.47	11.68	32.46	**0.005**	4.10	1.53	10.94	**0.001**	16.49	3.30	82.29	**<0.001**	77.04	9.25	641.4
Age (years-old)	80.54	**<0.001**	0.155	1.03	0.99	1.08	**0.004**	0.85	0.76	0.95	**<0.001**	0.78	0.70	0.88	0.972	1.00	0.91	1.09
Onset (years-old)	4.78	0.311	0.486	0.98	0.94	1.03	0.170	1.08	0.97	1.21	0.622	0.97	0.86	1.09	0.829	1.01	0.93	1.10
Studies (university)	23.66	**<0.001**	**<0.001**	0.28	0.15	0.51	0.403	0.59	0.17	2.04	0.195	0.32	0.06	1.79	0.579	1.37	0.45	4.21
Civil status (married)	3.37	0.498	0.144	0.71	0.45	1.12	0.392	0.68	0.28	1.64	0.152	0.37	0.09	1.44	0.633	0.80	0.31	2.03
TCI-R: Novelty seeking	56.44	**<0.001**	**<0.001**	0.96	0.95	0.98	**<0.001**	0.92	0.89	0.94	**<0.001**	0.90	0.87	0.93	**0.001**	0.95	0.92	0.98
TCI-R: Harm avoidance	16.04	**0.003**	**<0.001**	0.97	0.95	0.99	**0.002**	0.96	0.93	0.98	**0.008**	0.96	0.94	0.99	**0.028**	0.97	0.94	1.00
TCI-R: Reward dependence	5.78	0.217	0.521	0.99	0.98	1.01	0.515	0.99	0.96	1.02	0.432	0.99	0.96	1.02	0.106	1.03	0.99	1.07
TCI-R: Persistence	31.17	**<0.001**	0.733	1.00	0.98	1.01	**<0.001**	0.96	0.93	0.98	**0.002**	0.96	0.94	0.99	0.223	0.98	0.96	1.01
TCI-R: Self-directedness	12.17	**0.016**	**0.018**	0.98	0.97	1.00	**0.010**	0.97	0.95	0.99	**0.046**	0.97	0.95	1.00	**0.003**	0.96	0.93	0.99
TCI-R: Cooperativeness	3.29	0.511	0.740	1.00	0.98	1.01	0.340	1.02	0.98	1.05	0.504	1.01	0.98	1.04	0.445	0.99	0.95	1.02
TCI-R: Self-Transcendence	3.55	0.470	0.703	1.00	0.98	1.01	0.219	1.02	0.99	1.05	0.611	1.01	0.98	1.04	0.724	0.99	0.96	1.03

*Bold, significant coefficient. Models obtained with multinomial regression entering simultaneously the set of predictors (ENTER procedure) (McFadden-R^2^= 0.283)*.

### Predictive models of psychopathology symptoms for the CBB group

Table 4 contains the three multiple regressions measuring the predictive capacity of the patients' sex, age, age of onset, and personality traits profile on levels of depression, anxiety, and GSI-index measured through the SCL-90-R for the CBB group (*n* = 110). High levels of depression were associated with women and patients with high scores in novelty seeking, harm avoidance, and cooperativeness, but low levels in reward dependence and self-directedness. High anxiety was registered for women, and those patients with high scores in harm avoidance and low scores in self-directedness. High GSI scores were linked to women; obtaining high scores in novelty seeking, harm avoidance and self-transcendence; and low scores in self-directedness.

**Table 4:** **Predictive capacity of age, age of onset, and personality traits in the psychopathology symptom levels for the CBB group (***n*** = 110)**.

**Dependent variable →**	**SCL-90-R: depression** ***(adjusted R**^**2**^**= 0.601)***							**SCL-90-R: anxiety** ***(adjusted R**^**2**^**= 0.580)***							**SCL-90-R: GSI index** ***(adjusted R**^**2**^**= 0.676)***						
**↓ Predictor**	**B**	**SE**	**Beta**	**t**	***p***	**95%CI(B)**		**B**	**SE**	**Beta**	**t**	***p***	**95%CI(B)**		**B**	**SE**	**Beta**	**t**	***p***	**95%CI(B)**	
Constant	0.439	1.869		0.235	0.815	−3.28	4.16	−2.562	1.870		−1.370	0.174	−6.28	1.16	−1.900	1.339		−1.419	0.160	−4.56	0.76
Sex (male)	−0.692	0.172	−.289	−4.032	**<0.001**	−1.03	−0.35	−0.516	0.172	−0.221	−3.005	**0.003**	−0.86	−0.17	−0.469	0.123	−0.246	−3.817	**<0.001**	−0.71	−0.22
Age (years)	−0.004	0.016	−.041	−0.266	0.791	−0.04	0.03	−0.006	0.016	−0.059	−0.369	0.713	−0.04	0.03	−0.004	0.011	−0.047	−0.338	0.736	−0.03	0.02
Age of onset (years)	0.001	0.014	0.006	0.037	0.970	−0.03	0.03	0.010	0.014	0.114	0.718	0.474	−0.02	0.04	0.005	0.010	0.069	0.492	0.624	−0.02	0.03
TCI−R: Novelty seeking	0.014	0.006	0.180	2.167	**0.033**	0.00	0.03	0.012	0.006	0.167	1.959	0.053	0.00	0.02	0.013	0.004	0.210	2.809	**0.006**	0.00	0.02
TCI−R: Harm avoidance	0.019	0.006	0.356	3.436	**0.001**	0.01	0.03	0.025	0.006	0.479	4.504	**<0.001**	0.01	0.04	0.023	0.004	0.522	5.591	**<0.001**	0.01	0.03
TCI−R: Reward depend.	−0.012	0.005	−0.177	−2.193	**0.031**	−0.02	0.00	0.001	0.005	0.009	0.112	0.911	−0.01	0.01	−0.003	0.004	−0.052	−0.718	0.475	−0.01	0.01
TCI−R: Persistence	0.004	0.004	0.086	1.065	0.290	0.00	0.01	0.007	0.004	0.134	1.613	0.110	0.00	0.02	0.005	0.003	0.120	1.645	0.104	0.00	0.01
TCI−R: Self−directedness	−0.023	0.005	−0.503	−4.489	**<0.001**	−0.03	−0.01	−0.015	0.005	−0.333	−2.897	**0.005**	−0.02	0.00	−0.012	0.004	−0.332	−3.289	**0.001**	−0.02	0.00
TCI−R: Cooperateness	0.013	0.006	0.193	2.182	**0.032**	0.00	0.02	0.002	0.006	0.030	0.326	0.746	−0.01	0.01	0.002	0.004	0.040	0.495	0.622	−0.01	0.01
TCI−R: Self−Transcend.	0.002	0.005	0.037	0.464	0.644	−0.01	0.01	0.009	0.005	0.149	1.815	0.073	0.00	0.02	0.008	0.004	0.157	2.180	**0.032**	0.00	0.02

*Models obtained with multiple regression entering simultaneously the set of predictors (ENTER procedure). Bold, significant coefficient*.

## Discussion

This study analyzed the specific characteristics of CBB compared to other behavioral addictions: gambling disorder, Internet gaming disorder, Internet addiction and sexual addiction. The results obtained in a large sample of treatment-seeking patients show that although CBB could likely be related to other addictive behaviors, significant differences in its phenomenology exist. CBB is characterized by a higher proportion of women, older age and age of onset, poorer general psychopathological state and higher levels of novelty seeking and harm avoidance and moderate levels of reward dependence, persistence, and cooperativeness. In this sense, CBB patients could be described as being curious, easily bored, impulsive and active seekers of new stimuli and reward, but at the same time showing pessimism and worry in anticipation of upcoming challenges. Several sociocultural contributors might also take part in the onset and maintenance of CBB, such as one's personal financial state, materialistic values, and the variety of goods available (Dittmar, 2005). One should also take into account the fact that in hoarding, one of the most commonly reported symptoms is acquiring behavior, and that other studies have identified numerous similarities between the two disorders (Frost et al., 2002). Clinical differences are lower compared to sex addiction and higher compared to gambling disorder, IGD, and Internet addiction.

Regarding gender, differences between diagnostic subtypes emerged in this study: the CBB group included a considerably higher proportion of women compared to other behavioral addictions. This result is consistent with other studies, which had also reported higher levels of compulsive buying in women (Fattore et al., 2014; Otero-López and Villardefrancos, 2014). Possible reasons for the elevated prevalence of women with CBB are most likely related to the higher frequency of shopping as a recreational activity in this group and other related socio-cultural factors (Maraz et al., 2015).

Results of this study also show that the proportion of patients attending our specialized unit for CBB treatment had a tendency to increase during the last decade, with a similar trend occurring for Internet, IGD and sexual addictions. However, these proportions of treatment-seeking patients were significantly lower compared to the number of consultations for gambling disorder. With regards to the evolution of the proportion of CBB consultations during the last decade, our results point to a drop between the years of 2010 and 2013, coinciding with the worst years of the economic crisis in Europe, and, more specifically, in Spain. Moreover, this decrease is consistent with results exploring other behavioral addictions requiring substantial amounts of money. In the case of gambling disorder, a significant drop in prevalence was also found during the European economic crisis (Jiménez-Murcia et al., 2014b), especially in 2010.

Patients' age and the mean age of onset of problematic addictive behaviors greatly differed between diagnostic subtypes, with older ages being found in CBB (mean age was 43.3 years and mean onset 38.9, nearly followed by gambling disorder and sex addiction) and younger ages for IGD (mean age 22.0 and mean onset 19.9 in this study). This finding dovetails with several studies reporting that young age is linked to problematic video game and Internet use (Griffiths and Meredith, 2009; Achab et al., 2011; Jiménez-Murcia et al., 2014a). Other variables, such as the endorsement of materialistic values among young people, should be considered in the scientific literature as an effective mediator of the young age of onset in some addictive behaviors, particularly in the case of compulsive buying (Dittmar, 2005).

Differences in the psychological state and personality traits between the diagnostic subtypes are also relevant: CBB and sexual addiction showed similar profiles, with their psychopathological symptoms and personality scores being clearly worse than for gambling, IGD, and Internet addictions. Although in behavioral addictions, impulsivity appears to be a core feature (Dell'Osso et al., 2006; Billieux et al., 2012; Lorains et al., 2014), multiple studies also show the existence of high levels of compulsivity (Blanco et al., 2009; Fineberg et al., 2010; Bottesi et al., 2015). Impulsivity and compulsivity seem to be characterized by deficits in self-control capacity. Nonetheless, a key distinction between impulsivity and compulsivity is that the former is associated with immediate gratification and reward seeking, while compulsion is aimed at finding relief from negative emotions.

Overall, the findings obtained in this study show that this combination of symptoms (impulsive/compulsive) is especially prominent in CBB and sexual addiction. This leads us to postulate the existence of phenotypical and possibly endophenotypical overlap across these disorders. This results support previous research that has found numerous shared features in CBB and sexual addiction (Müller et al., 2015a) and other behavior addictions (Lejoyeux et al., 2008; Villella et al., 2011). However, a notable difference in the sex prevalence of both disorders (higher proportion of women in CBB and of men in sex addiction) exists. This fact may partly explain why the similarities between these disorders have hardly been explored (Álvarez-Moya et al., 2007). Lastly and quite possibly due to higher awareness of this condition, the number of GD patients was vastly higher than the other behavioral addictions examined in this study. Future studies should aim to use larger, more diverse samples in order to overcome this drawback. The role of materialistic values and hoarding are also topics that should be considered. However, our findings should be considered in light of their limitations and we stress that the features of treatment-seeking patients in a single unit for behavioral addictions does not necessarily reflect the actual frequency of an addiction in the origin population. The lack of consensus regarding the diagnostic criteria for the behavioral additions examined in the study also limits the generalizability of our results.

## Conclusion

The results of this study suggest that CBB should be considered as a behavioral addiction, in the same manner as other excessive behaviors (such as sexual addiction, gambling, IGD, or Internet addiction). At present, an integrative model for describing the underlying mechanisms which lead to the onset and development of the CBB is not available. Additional empirical evidence is needed to identify core contrasting factors so as to clarify whether CBB represents a distinct psychiatric entity or is better conceptualized as an epiphenomenon of other psychiatric disorders characterized by addictive and/or impulse control behaviors. As with most complex, multifaceted-multidimensional processes, these studies should cover different areas: neurobiological (to recognize implicated regions, networks, and executive/cognitive functions), clinical (to dispose of the complete patient phenotype and to identify distinct developmental trajectories of the condition), and psycho-socio-cultural (to clarify what consumer-culture and financial resources interact with psychological, individual, and personality traits to lead to an increase in buying behavior).

Ultimately, a detailed understanding of the CBB will allow for improving prevention and treatment efforts. New empirical studies are required to gain a better understanding of the etiology of CBB and to establish more effective intervention programs.

## Author contributions

RG, FF, JM, ST, and SJ designed the experiment based on previous results and clinical experience of AD, MB, LM, NA, NM, and MG. RG, GM, TS, FF, and SJ conducted the experiment, analyzed the data, and provided a first draft of the manuscript. SJ, TS, GM, RG, and FF further modified the manuscript.

## Funding

This manuscript and research was supported by grants from Instituto de Salud Carlos III (FIS PI11/00210, FIS14/00290, CIBERObn, CIBERsam, and Fondos FEDER) and PROMOSAM (PSI2014-56303-REDT). CIBERObn and CIBERSAM are both an initiative of ISCIII. This study was cofunded by FEDER funds/European Regional Development Fund (ERDF)—a way to build Europe and by a Ministerio de Economía y Competitividad grant (PSI2015-68701-R).

### Conflict of interest statement

The authors declare that the research was conducted in the absence of any commercial or financial relationships that could be construed as a potential conflict of interest.
